# Reliability and reproducibility of CBCT assessment of mandibular changes before and after treatment for Class III growing patients – an easy and quick way for evaluation

**DOI:** 10.1186/s12887-023-04404-4

**Published:** 2023-11-28

**Authors:** XiaoYing Hu, Gary Shun Pan Cheung, YiYang Zhang, RuoNan Sun, FuSheng Dong

**Affiliations:** 1 Department of Dental Surgery, The University of Hong Kong-Shenzhen Hospital, Shenzhen, 518000 China; 2 Stomatology Student, School of Basic Medical Sciences, Hebei University, Hebei, 050000 China; 3 Dentistry Department, Shijiazhuang City Second Hospital, Hebei, 050000 China; 4 College of Stomatology, Key Laboratory, Hebei Medical University, Hebei, 050017 China

**Keywords:** Reliability, Reproducibility, 3-dimensional mandibular Changes, Class III growing patients, Voxel-based superimposition, CBCT

## Abstract

The objective of this study was to evaluate intraobserver reliability and inter-observer reproducibility of a 3-dimensional (3D) assessment method for mandibular changes of growing patients after orthodontic treatment for Class III malocclusion.

**Methods** Cone-beam computed tomography (CBCT) scans were performed before and after orthodontic treatment for 27 patients. During the scan, the patient was positioned such that his/her mandibular plane was parallel to floor. Three observers independently worked on the DICOM data, reconstructed the pre- and post-treatment 3D models in software, selected the stable anatomical structures (basal bone area from the lingual surface of the symphysis to the distal aspect of the first molars) to guide the automated superimposition process. Then, each observer registered 14 anatomical landmarks on the virtual models, for three times after suitable interval, to generate 3 sets of coordinates; the mean was taken as the coordinates for that particular landmark. The intraobserver reliability and inter-observer reproducibility of the method were analyzed.

**Results** The ICCs was > 0.90 for 25 (92.6%) of the intraobserver assessments. The precision of the measurement method was < 0.3 mm in 24 (88.9%) cases. The interobserver reproducibility errors were < 0.3 mm in 21 of the 27 cases.

**Conclusions** The intraobserver reliability and inter-observer reproducibility of 3D assessment of mandibular changes using the virtual models were excellent.

## Introduction

Mandibular growth has always been a popular topic in clinical research since the introduction of cephalometric analysis to the field of orthodontics. Compared to the study of maxillary growth, study of the mandible is more complex and difficult, due to its mobility and variation of location in relation to the skull base. Traditionally, cephalograms before and after treatment were superimposed to reveal the effect of orthodontic treatment and of mandibular growth. The superimposition must be guided by making reference to stable anatomical structures that can be identified on the cephalograms, such as the mandibular canal and inferior border of the mandible [1, 2]. However, as most patients with skeletal malocclusion are associated with facial asymmetry [3], a simple sagittal assessment using 2-dimensional (2D) lateral cephalogram would not fully reflect the three-dimensional (3D) changes in the size, shape and relative location of the mandible. With the advent of imaging technology, particularly the introduction of cone-beam computed tomography (CBCT) in dentistry, 3D cephalometric analysis has become a valuable tool for clinical research, as well as orthodontic treatment planning. The diagnostic accuracy of CBCT outweighs that of 2D radiography, especially in the assessment of labiopalatal direction and evaluation of the contact relationship between adjacent teeth [4]. Superimposition of virtual models for making 3D measurements is much more complex than with 2D images, but that would provide a more comprehensive analysis of the changes in size and position of the structure in 3 dimensions [5]. Despite the difficulty, some research has been conducted in this area and progress has been made [6, 7]. Lagravère and coworkers [8] put forward an ELSA standardized coordinate system based on 4 landmarks: (i) midpoint between the geometric centers of the foramina spinosum; (ii & iii) the right (rSLEAM) and left superior-lateral border of the external auditory meatus (lSLEAM); and (iv). the mid-dorsum foramen magnum (MDFM). However, because the skull base is situated far away from the facial area, any discrepancy in mapping the mandible to the coordinate system would be magnified that might incorporate errors in any 3D superimposing measurements to a clinically significant level, especially when the mandible is concerned. Leonardi et al. [9] evaluated the mandibular asymmetry in youngsters with posterior unilateral crossbite, through cone-beam computed tomography and a reverse engineering software. That was a surface-based superimposition and the software tended to smoothen any irregular surfaces, which algorithm tended to distort the original surface of the CBCT scan and, hence, might introduce unnecessary errors.

Studying the growth of the mandible relies on measurements made to stable locations in the skull or the mandible itself. The latter might be preferred, due to the mobility of the mandible and its distance from the skull base, especially for the chin and symphysis area. One study of the mandibular growth was done by implanting bone plates and screws at presumably stable anatomical areas, prior to CBCT and 3D superimpositions [10]. Certain mandibular structures (chin and symphysis region) were identified as stable in growing individuals, and thus such implants may no longer needed to quantify mandibular growth, as well as treatment changes.

In the present study, we evaluated the 3-dimensional mandibular changes in growing patients, who had received orthodontic treatment, for for Class III malocclusion and examine the intraobserver reliability and inter-observer reproducibility of a 3D voxel-based superimposition method..

## Material and methods

The material for this study consisted of pairs of pre- and post-treatment CBCT scans of 27 children, aged 8 to 11 years, who had received protraction therapy for their Class III malocclusion at the Department of Orthodontics of the Hebei Medical University Stomatological Hospital. The two scans were taken on average 24 to 26 months apart by the same radiologist. The inclusion criteria were: 1) cervical vertebral maturation (CS1-CS3); 2) no discernible craniofacial asymmetry; 3) no discernible mandibular asymmetry; 4) no temporomandibular joint disorders; 5) no history of maxillofacial trauma or surgery in the region; 6) no systemic disease; and 7) clear and legible 3D images. The study protocol was approved by the Medical Ethics Commission of the Hebei Medical University, with all patients or their parents giving informed consent.

**Fig. 1:**
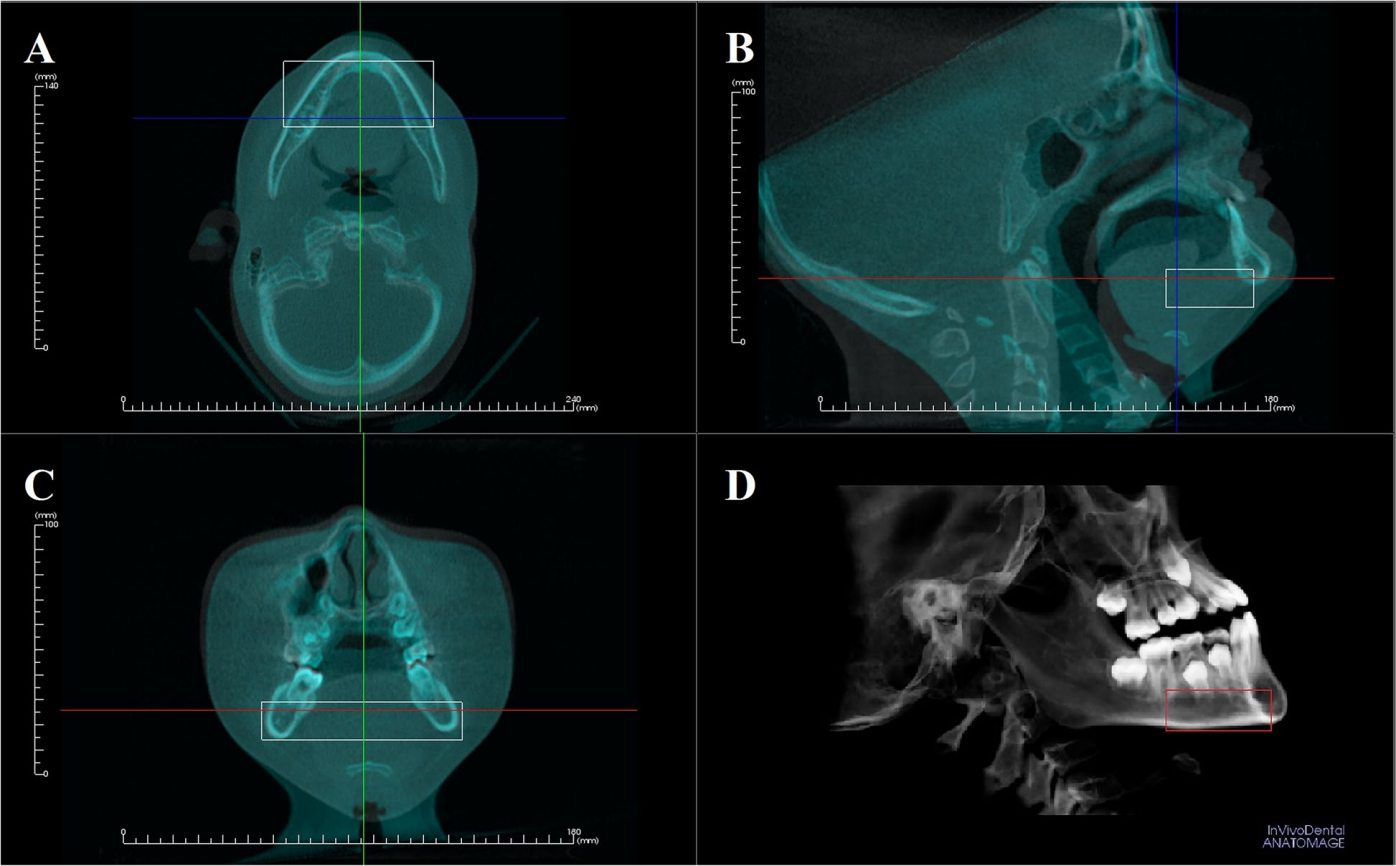
Superimposition of image guided by the region selected in the boxed volumes. **A **axial; **B** sagittal; **C **coronal; **D **3Dview

The parameters of the CBCT machine (Dental Volumetric Tomograph, KaVo 3D eXam, Imaging Sciences International LLC, Hatfield, PA, USA) were set at 120 kVp, 18.54 mA, 23 × 17 cm field of view, 0.3 mm voxel size, and scan time of 8.9 s. The preoperative scans were taken in the routine manner with Frankfurt plane oriented horizontally. The post-treatment scan was done with the patient’s mandibular plane oriented parallel to floor (Fig. 1). Data were exported in DICOM format into a 3D imaging software (InvivoDental software 5.1.3, Anatomage Inc, San Jose, USA), in which a virtual model of the patient was created in a three-dimensional coordinate system. The software offered a “superimposition” module. To achieve fully automated voxel-based superimposition of the pre- and post-treatment scan, some stable anatomical area had to be defined first. This was done by clicking on the "voxel registration" button, which called up 3 boxes in the sagittal, coronal and axial view for the observer to select the stable region to guide the superimposition [11] – this region being the basal bone area extending from the lingual surface of the symphysis to the distal aspect of the first molars (Fig. 1). After clicking on the "start" button and the automatic voxel-based superimposition of the 3D images began. The whole process of registration and superimposition took approximately 3 min.

**Fig. 2:**
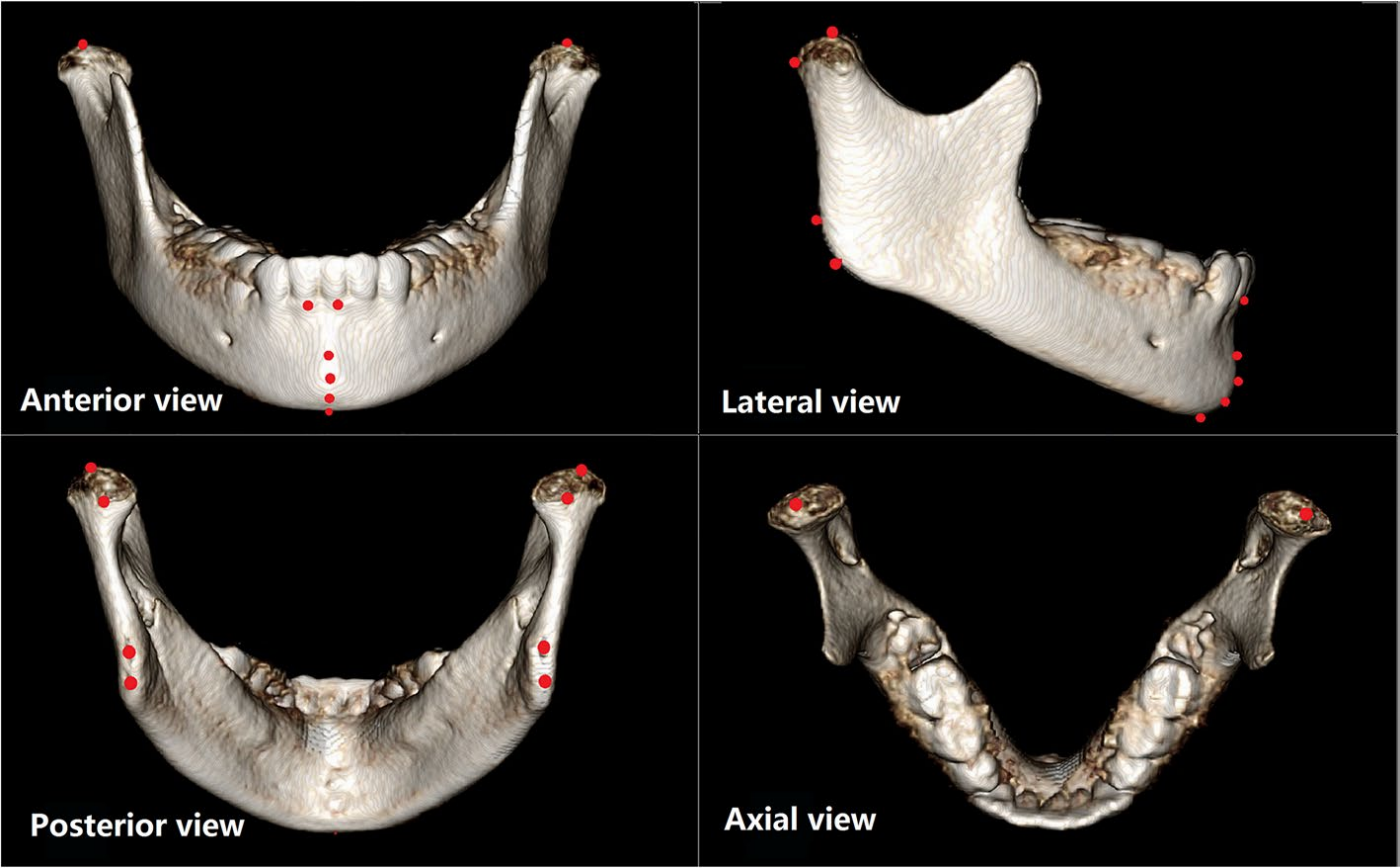
Landmarks displayed in the 3D virtual surface model

A total of 14 landmarks [11–13] (or 9 in total, if the left and right were considered as contributing to one landmark) were located by each observer independently on every surface-rendered 3D model (Fig. 2), according to defined criteria for each (Table 1). To identify the landmarks, the observer would navigate to any (sagittal, coronal or axial) view and select the most appropriate slice in that view for registration. The three-dimensional coordinates of each landmark were then automatically recorded by the software. For those landmarks that might be difficult to define in a special view, the observer could call up the surface-rendered model to assist with the localization (see Fig. 2). After all 14 landmarks were registered, the values of their X, Y and Z coordinate were exported into a spreadsheet (Microsoft Excel, Microsoft Corporation, Redmond, WA, USA) for analysis later.

**Table 1:** 14 Landmarks selected for the study

Landmark name	Anatomic region	Lateral view	Axial view	Anteroposterior view
1. Left/Right lower incisal alveolar ridge (lLIAR) / (rLIAR)	Premandbula alveolar	Anterior–Superior–most point	Anterior-most point	Middle point in the nteroposterior slice determined by the lateral and axial view
2. B point (B)	Anterior surface of the mandibular symphysis	Posterior-most point	Middle-anterior–most point on the anterior contour	Middle point in the anteroposterior slice determined by the lateral and axial views
3. Pogonion (Po)	Contour of the bony chin	Anterior-most point	Middle-anterior–most point on the anterior contour	Middle point in the anteroposterior slice determined by the lateral and axial views
4. Gnathion (Gn)	Contour of the bony chin	Anterior-inferior–most point	Middle-anterior-inferior–most point	Inferior-most point
5. Menton (ME)	Lower border or the mandible	Inferior-most point	Middle-inferior–most point	Inferior-most point
6. Left /Right mandibular gonion(lGo)/ (rGo)	Angle of the left/ right mandibular body	Middle point along the angle	Posterior-most point	Inferior-most point
7. Left/Right ramus point (lRP)/(rRP)	Posterior border of the left/ right mandibular ramus	Middle-posterior–most point between the condylar neck and the angle of the mandibular body	Middle-posterior–most point	Inferior-most point
8. Left/ Right condylion (lCo)/ (rCo)	Left /Right condyle	Superior-most point	Middle point in the axial slice level determined by the lateral and anteroposterior views	Middle-superior–most point
9. Left/Right posterior mandibular condyle(lPCo)/ (rPCo)	Left//Right condyle	Posterior-most point	Middle-posterior–most point	Middle point in the anteroposterior slice determined by the lateral and axial view

Three observers (an orthodontist, a postgraduate orthodontic student, and a dental radiologist) were trained and calibrated for the entire registration, superimposition and registration process by using a set of 30 CBCT scans not included in this study. Then, the 3 observers worked independently throughout, to define the 14 anatomical landmarks Table 1 in the superimposed CBCT volume (from before and after treatment for the same patient), as described above. This process took the observers about 3 to 5 min to complete. Each observer repeated the process 2 more times at 5-day intervals, and the mean X, Y, Z coordinates from the three attempts were taken as the registration of the corresponding landmark by that observer.

At the beginning of the experiment, we planned to collect CBCT data of 30 children before and after orthodontic treatment. However, in the later voxel-based superimposition, it was found that the head pose of CBCT data did not meet the requirements, so 3 cases were excluded and finally 27 cases were included in the study.

Statistical analyses were done in a statistical software package (SPSS version 21.0; SPSS, Chicago. IL, USA). The first step was to calculate the difference in the value of the coordinates (i.e. changes arising from treatment and growth) for each landmark – this was done by subtracting the pre- and post-treatment coordinate values of that landmark. Then, the intraobserver agreement was estimated by computing the Intraclass correlation coefficients (ICCs). A primary evaluation of the normal distribution and homogeneity test of variance of the data was performed with Shapiro–Wilk normality test and Levene’s test. Paired t-test was used for intraobserver reliability assessments, and unpaired t-test and Mann–Whitney U-test were used for inter-observer comparisons.

## Results

Using voxel-based superimposition, the morphological skeletal changes of the mandible was revealed for these Class III patients before and after protraction therapy. The intraobserver agreement was estimated by the ICCs for the differences in the X, Y and Z coordinate of each landmark. The ICCs results were listed in Tables 2 and 3. In general, the ICC indicated excellent reliability for intraobserver assessments. Table 2 listed the reliability estimated by the ICC for the coordinate difference of each landmark. Low ICC scores of two bilateral landmarks showed relatively poor reliability: the X/Z coordinate of the right and left condylion, Table 3 showed the frequency of the intraobserver reliability estimated by th ICC for the X, Y, and Z coordinates. The ICCs was > 0.90 for 25 (92.6%) cases, of which excellent reliability (ICC > 0.95) was noted in 19 (70.4%) cases of the intraobserver assessments and 6 cases (22.2%) showed good reliability (0.90 ≤ ICC < 0.95).

**Table 2:** Assessment of intraobserver reliability: (a) Intraclass correlation Coefficient (ICC) and confidence interval for repeated measurements (from the triplicate evalation)

	X	Y	Z	X	Y	Z
Landmark	Intraclass Correlation			95% Confidence Interval		
Right lower incisal alveolar ridge (rLIAR)	0.97	0.98	0.98	0.91–0.99	0.94–1.00	0.92–1.00
Left lower incisal alveolar ridge (lLIAR)	0.96	0.97	0.99	0.89–0.98	0.91–1.00	0.94–1.00
B point (B)	0.95	0.96	0.98	0.90–0.98	0.89–0.98	0.91–1.00
Pogonion (Po)	0.95	0.98	0.98	0.86–0.98	0.90–1.00	0.90–1.00
Gnathion (Gn)	0.94	0.97	0.98	0.86–0.97	0.91–0.99	0.92–1.00
Menton (ME)	0.96	0.99	0.98	0.89–0.99	0.90–1.00	0.90–0.99
Right mandibular gonion (rGo)	0.97	0.86	0.91	0.91–0.99	0.82–0.95	0.88–0.97
Left mandibular gonion (lGo)	0.98	0.88	0.90	0.94–0.99	0.84–0.96	0.85–0.98
Right ramus point (rRP)	0.96	0.48	0.87	0.88–0.98	0.35–0.74	0.84–0.97
Left ramus point (lRP)	0.95	0.53	0.90	0.86–0.98	0.41–0.81	0.86–0.98
Right condylion (rCo)	0.57	0.98	0.44	0.47–0.86	0.91–0.99	0.31–0.67
Left condylion (lCo)	0.55	0.96	0.48	0.44–0.82	0.89–0.99	0.35–0.76
Right posterior mandi- bular condyle (rPCo)	0.95	0.97	0.97	0.87–0.98	0.91–0.99	0.91–1.00
Left posterior mandi -bular condyle (lPCo)	0.94	0.95	0.98	0.85–0.97	0.88–0.98	0.92–1.00

**Table 3:** Assessment of intraobserver reliability:(b) ICC values of changes in madibular measurements difference in X, Y, and Z coordinates

Range	Coordinate difference							
	X		Y		Z		Total	
	n	%	n	%	n	%	n	%
ICC ≥ 0.95	10	71.4	10	71.4	8	57.1	28	66.7
0.90 ≤ ICC < 0.95	2	14.3	0	0	3	21.4	5	11.9
0.85 ≤ ICC < 0.90	0	0	2	14.3	1	7.1	3	7.1
0.80 ≤ ICC < 0.85	0	0	0	0	0	0	0	0
ICC < 0.80	2	14.3	2	14.3	2	14.3	6	14.3
Total	14	100.0	14	100.0	14	100.0	42	100.0

To assess the reproducibility of this measurement method, Table 4 showed the frequency counts of the the difference in mean values of the coordinate change arising from treatment and growth for each landmark. The precision of the measurement was better than 0.3 mm in 24 (88.9%) cases. For interobserver reproducibility estimated by the ICC for the difference in mean values for the X, Y, and Z coordinates, similar results were obtained – the interobserver reproducibility errors were < 0.3 mm in 21 of the 27 cases; only 6 cases (22.2%) showed error ≥ 0.3mm.

**Table 4:** Frequency of the difference in mean values for difference among measurement (mm) and for reproducibility of the measurement method (mm)

	**Among measurement**								**Reproducibility of the measurement method**							
***Range (mm)***	***Coordinate difference***								***Coordinate difference***							
	***X***		***Y***		***Z***		***Total***		***X***		***Y***		***Z***		***Total***	
	***n***	***%***	***n***	***%***	***n***	***%***	***n***	***%***	***n***	***%***	***n***	***%***	***n***	***%***	***n***	***%***
**≥ 0.3**	**2**	**-14.3**	**4**	**-28.6**	**6**	**-42.9**	**12**	**-28.6**	**2**	**-14.3**	**4**	**-28.6**	**7**	**-50**	**13**	**-31**
**0.15≤ x < 0.3**	**11**	**-78.6**	**6**	**-42.9**	**4**	**-28.6**	**21**	**-50**	**12**	**-85.7**	**8**	**-57.1**	**4**	**-28.6**	**24**	**-57.1**
**≤ 0.15**	**1**	**-7.1**	**4**	**-28.6**	**4**	**-28.6**	**9**	**-21.4**	**0**	**0**	**2**	**-14.3**	**3**	**-21.4**	**5**	**-11.9**
**Total**	**14**	**-100**	**14**	**-100**	**14**	**-100**	**42**	**-100**	**14**	**-100**	**14**	**-100**	**14**	**-100**	**42**	**-100**

## Discussion

There are several methods and software available nowadays for superimposition and measurement of 3D data. Voxel-based registration and surface-based registration are common approaches. Almukhtar et al. [14] compared the 3D stacking accuracy of surface-based versus voxel-based registration, and reported that voxel-based registration showed a low variability in the mean distance between corresponding surface landmarks when compared to surface-based registration, especially for landmarks situated on soft tissues. Voxel-based approach assesses the entire selected volume and the grey scale difference of the voxels to align the two DICOM scans for the best superimposition. InvivoDental software and Dolphin 3D and Mimic are examples of software that are frequently used to perform voxel-based registration [15]. Our CBCT machine came equipped with InvivoDental 5.1.3 software, and so we used it in this study for convenience. The purpose of this study was not to compare different software brands, but to evaluate the reliability of the method to assess mandibular changes for growing patients in 3 dimensions, by examining (the coordinates of) the landmarks before and after the treatment [16], as measured by different observers. The whole 3D measurement analysis was conducted in 6 steps: CBCT scan, digital model construction, model reorientation, voxel-based superimposition, registration of landmarks, and quantitative measurement. The software automatically created the coordinate system while constructing the models. Although the coordinate system generated for each CBCT scan would vary with the head position and orientation during the scan, the virtual 3D model must be reoriented by making reference to stable anatomical structures before superimposition. Once the voxel-wise rigid registration of the reference area was completed, the superimposition process was fully automatic by the software. The stable reference anatomy in the mandible selected was the region from the lingual surface of the symphysis to the distal aspect of the first molars at the level of basal bone [11].

Unlike the 2-dimensional cephalometric analysis, 3D cephalometric measurements involved bilateral structural landmarks. Altogether, 14 landmarks were selected for this study (see Fig. 2): Right lower incisal alveolar ridge (rLIAR); Left lower incisal alveolar ridge (lLIAR); B point (B); Pogonion (Po); Gnathion (Gn); Menton (Me); Right mandibular gonion (rGo); Left mandibular gonion (lGo) [13]; Right condylion (rCo); Left condylion (lCo) [11]; Right ramus point (rRP); Left ramus point (lRP); Right posterior mandibular condyle (rPCo); Right posterior mandibular condyle (lPCo) [12]. Stable reference points on the mandible itself were chosen to avoid any additional error due to mandible movement or measurement to distant structures.

Errors in this research might occur in any of the following 4 steps of the overall process: a) CBCT scan, b) the model reorientation, c) the voxel-based superimposition, and d) landmark registration. CBCT scans, typically, are commonly taken with different head position [17], which may lead to inter-observer difference during scan approximation. Therefore, it is preferable to standardize the head position at the time of the scan. In this study, the head position requirements for the post-treatment scan were as follows: both sides of the mandibular body and mandibular ramus would overlap as much as possible; and the mandibular plane was parallel to floor. As the great majority of landmarks to be recorded by the software (except rLIAR and lLIAR) were situated on a curved surface of the mandible. Therefore, model reorientation is the key to localisation of the landmarks. As the craniofacial characteristics of the patients included in this study were basically symmetrical, the difficulty and error of model reorientation were relatively small. The voxel-based superimposition may be considered as semi-automated – first, the observers selected the area; then the computer automatically superimposed the images. Errors in this step may be related to the (wrong) area being selected by the observer. If the region selected on the pre- and post-treatment model should differ, the superimposition would generate erroneous results. To avoid this, each observer would continue to superimpose until the 2 superimposition results were consistent. Finally, there were 2 sources of error in the localization of landmarks in this study. The first one was related to the difficulty in choosing the best slice and region to identify the landmarks. Second, the 3 planes were interrelate such that adjusting the slice in one plane would result in movement of the reference lines in other planes. Therefore, some experience on the part of the observers was essential and, hence, training on a sizeable sample was done beforehand for the observers. In the present study, although the 3 observers had different working backgrounds, that seemed to have little impact on the aount of errors in measurement. Training and calibration of the observers or assessors cannot be overemphasized in any studies. On the other hand, some other factors related to the precision and reproducibility of the 3D measurement analysis should benefit from further investigation, such as the accuracy of the CBCT [18], voxel size [19], scanning time, and scanning range. The voxel size used in this study was 0.3 mm. Recent studies emphasized that a smaller voxel size will result in better measurement accuracy, to decrease the measurement errors [19].

Overall, the present results indicated a fairly good intraobserver reliability and inter-observer reproducibility of this superimposition and measurement method. The less-than-perfect level of reproducibility might be explained by ambiguous definition criteria of some landmarks and reliance of the observers to be able to select the best perspective and slice to reveal the landmarks. In addition, it can be noticed in Table 3 that the reliability of the Z coordinate definition was inferior to that of the X and Y coordinates, which could be related to inconspicuous appearance of some landmarks on the Z plane. Therefore, the choice of landmarks could have an impact on the intraobserver reliability, as well as inter-observer reproducibility of the measurements [20].

In summary, we verified a 3D quantitative measurement method for the assessment of mandibular changes for growing patients who had undergone protractive therapy. Each calibrated observer spent a total of 6 to 8 min to finish all the steps. Clinically, this voxel-based superimposition methodmight be useful, as the parents of the patient may be informed of the progress of orthodontic treatment quickly [21], in an effective and eficent manner, hihc would promote good doctor-patient communication.The doctors could show the changes of before and after treatments to the patients conveniently and efficiently. The landmarks selected for the study largely represented mandibular changes and are particularly useful for Class III malocclusions. ICCs were > 0.90 for 25 (92.6%) cases of intraobserver assessments (Table 4) and the precision of the measurement method was within 0.3 mm in 24 (88.9%) cases with interobserver errors also smaller than 0.3 mm in 21 of the 27 cases (Table 4). Overall, the intraobserver reliability and inter-observer reproducibility of this measurement method were regarded as excellent [22, 23]. New landmarks may be explored and tested to see whether this method further refined or be applicable to other treatment or growth assessment.

## Conclusion

The voxel-based superimposition method showed excellent intraobserver reliability and inter-observer reproducibility for the assessment of mandibular changes before and after protraction therapy for Class III patients. It was easy and quick to evaluate the changes for growing children, and may be carried at the chairside.

## Data Availability

The datasets used and/or analysed during the current study available from the corresponding author on reasonable request.
